# Climate change and the increase of human population will threaten conservation of Asian cobras

**DOI:** 10.1038/s41598-021-97553-4

**Published:** 2021-09-13

**Authors:** Mohammad Abdul Wahed Chowdhury, Johannes Müller, Sara Varela

**Affiliations:** 1 Museum für Naturkunde, Leibniz-Institut für Evolutions- und Biodiversitätsforschung, 10115 Berlin, Germany; 2 Institut Für Biologie, Humboldt-Universität zu Berlin, 10115 Berlin, Germany; 3 Department of Zoology, University of Chittagong, Chattogram, 4331 Bangladesh; 4 Department of Medicine, Venom Research Centre, Chittagong Medical College, Chattogram, 4203 Bangladesh; 5 Departamento de Ecoloxía e Bioloxía Animal, Centro de Investigación Mariña, Universidade de Vigo, Grupo GEA, 36310 Vigo, Spain

**Keywords:** Climate-change ecology, Conservation biology, Ecology, Zoology, Ecology

## Abstract

Asian cobras (genus *Naja*) are venomous snakes distributed from the Middle East to Southeast Asia. Because cobras often live near humans settlements, they are responsible for a large part of snakebite incidents and as such pose a challenge for public health systems. In the light of growing human populations, correctly mapping the present and future ranges of Asian cobras is therefore important for both biological conservation and public health management. Here, we mapped the potential climatic niches of ten Asian cobra species for both the present and the future, with the aim to quantify changes in climate and human population densities relative to their current and future ranges. Our analyses reveal that cobras that are adapted to dry climates and inhabit islands have narrow climatic niches, while those of mainland species with larger geographic ranges are much wider. We also found a higher degree of fragmentation of future cobra distributions; within the next 50 years, Asian cobras will lose an average of around 60% of their current suitable climatic range. In the near future, *Naja mandalayensis*, *N. sputatrix*, *N. samarensis*, and *N. philippinensis* are likely to have no accessible suitable climate space left. Besides, a further increase of human populations in this region may also exponentially accelerate the effects of anthropogenic impacts. Solutions for conservation may involve awareness and appropriate use of law to overcome the rate of habitat degradation and the increase of animal trade of Asian cobras, while promoting investment on health systems to avoid snakebite fatalities.

## Introduction

Snakes are ectothermic predators that are declining globally^1,2^. Degradation of natural habitats is considered the main responsible factor for the decline^2^, which involves geographic shifts caused by climate change, and human impact resulting in the gradual extinctions of local populations^3–6^, leaving snake species vulnerable due to their poor dispersal ability^7^. In particular the effects of climate change have been recognized from diverse perspectives, such as changes in activity patterns^8^, alterations of phenotypic traits^9^, and decreases in range size^10^.

Cobras, genus *Naja*, are a clade within the front-fanged venomous snake family Elapidae that includes four subgenera, *Afronaja*, *Boulengerina, Uraeus*, and *Naja*. *Naja* is a monophyletic taxon generally considered to have originated in Africa^11,12^, whereas today 11 *Naja* species are found in Asia^11,13^. Of those, six are mostly distributed in mainland Asia and the remaining five are largely restricted to maritime Southeast Asia^14,15^. Most Asiatic *Naja* species have moderate to large ranges^14,16,17^; around half of the species are threatened and about 90% of them have decreasing or unknown population trends^17^. From a conservation perspective, and similar to snakes in general (see above), habitat alteration is considered the main threat to cobras^17^.

Anthropogenic stressors on biodiversity are continuously increasing but vary by geographic region and species^18^. Generally, increasing human populations have prompted the unsustainable transformation of natural habitats through deforestation, expansion of urban areas and agricultural lands, and overexploitation of natural resources^19^. The effect is undoubtedly high in densely populated regions and is especially true for Asia, as this continent contains around 50% of the world's human population, with South and Southeast Asia having the world’s highest human population density and growth rate^20^. Particularly urban human populations are growing faster in the Asia–Pacific region than anywhere else^21^. Furthermore, people of China, South and Southeast Asia broadly use snakes as food and for traditional medicine^22,23^. The rapid growth of human populations in this region includes more consumers for traditional medicine, food and products made from cobras^23^; consequently, these snakes occupy the top of the exploitation list to satisfy domestic and international demands^22^. To control exploitation and trade, all eleven Asian cobra species have been added to the CITES appendix II^24^. However, formulating proper conservation strategies can be challenging in areas where local people show profound, long established prejudice against cobras and other snakes.

Venomous snakes have important socio-economic and public health impacts in tropical regions. Globally around 70% of snakebite fatalities occur in Asia^25^, with South and Southeast Asia holding a major portion of this burden^26,27^. A considerable segment of snakebite incidents are caused by cobras^28^. Cobras forage in human settlements and adjacent areas^15^, which leads to conflicts with humans, and increasing urbanization and land transformation rates in Asia^29^ are likely to increase the frequency of such conflicts^30^.

In this study, we aim to understand the effect of climate change and human population pressure on the geographic distributions of Asian cobras. We will provide potential climatic niche maps for Asian cobras in response to current and future global change scenarios, and we will analyse human population growth. The results will allow us to discuss potential conservation strategies for Asian cobras and also consider snake-human conflict, which is of significant concern for public health^31^.

## Results

**Figure 1:**
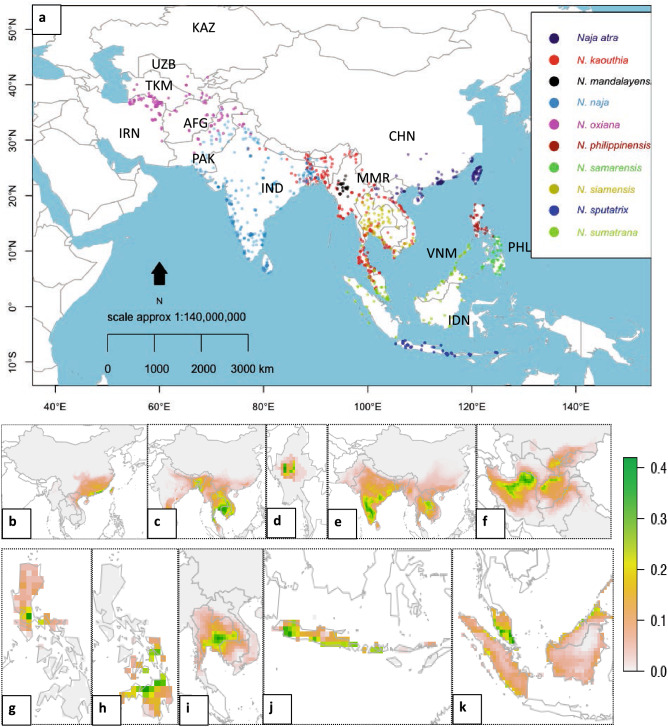
(**a**) The occurrence points of ten Asian cobras that are used in training the climate prediction model. The current potential climatic niche of ten Asiatic cobras that were produced by consensus of CCSM, CNRM, MIROC, and MIR global climate models (**b**) *Naja atra*, (**c**) *N. kaouthia*, (**d**) *N. mandalayensis*, (**e**) *N. naja*, (**f**) *N. oxiana*, (g) *N. philippinensis,* (**h**) *N. samarensis*, (**i**) *N. siamensis*, (**j**) *N. sputatrix*, and (**k**) *N. sumatrana*. The maps were generated from spatial polygon data frame of wrld_simpl function of maptools^64^ r-package.

**Figure 2:**
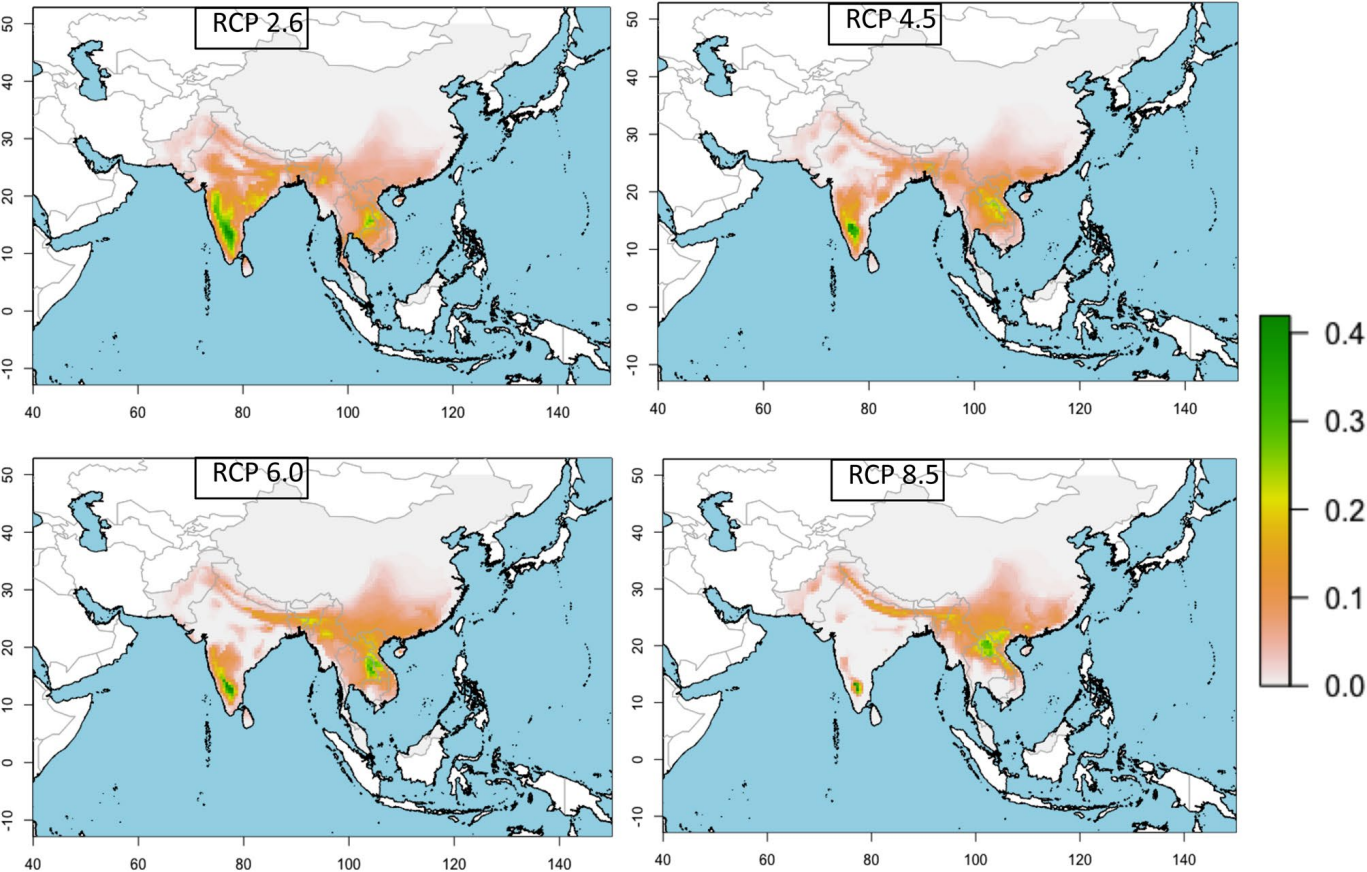
The consensus map of the future (in the year of 2070) suitable climatic niche of *Naja naja* in four different RCPs trajectories. The largest suitable climatic space is in RCP 2.6 and the least is in RCP 8.5. The maps were generated from spatial polygon data frame of wrld_simpl function of maptools^64^ r-package.

Overall, our results show that the present and future potential climatic niche of each studied cobra species is highly discontinued and fragmented (Figs. 1 and 2, Supplementary Figure). The species-level analysis reveals that the present climatic niche of *Naja naja* is the widest among all studied species, followed by *N. kaouthia* and *N. oxiana* (Fig. 1c,e,f). Currently, 14% of the total study area in Asia (present study area is 74,304,000 sq. km) is climatically suitable for *N. naja*, which spreads over the Indomalayan biogeographic realm (Fig. 1e). At the bottom of the ranking, four endemic cobras (*N. mandalayensis* in Myanmar^32^, *N. sputatrix* in Indonesia^33^, and *N. philippinensis* and *N. samarensis* in Philippines) have less than one million square kilometres of suitable climate surface (Fig. 1d,g,h,j). The last two species have the smallest niche, which is restricted to the Philippines (Table 1).

**Table 1:** Considering the RCP 6.0 scenario, the climatic niche size and human population in the range of the studied species at present time and in the next 50 years.

Species	Decrease in niche size (% of current niche)	Mean yearly decrease of the niche (× 1000 sq.km.)	Year to reach red list vulnerable (VU) EOO	Size of the three potential niche zones (million sq. km.)			Change in the human population (2020–2070) in three niche zones (No. /sq. km.)		
				1	2	3	1	2	3
*Naja atra*	− 40.14	− 20.09	2144	1.29	0.28	1.21	− 56	− 55	− 55
*N. kaouthia*	− 27.91	− 39.38	2199	2.19	0.22	4.86	23	− 16	− 16
*N. mandalayensis*	− 100.00	− 4.18	2065	0.21	0.00	0.00	− 21	♦	♦
*N. naja*	− 12.15	− 25.99	2431	1.74	0.44	8.96	8	− 33	− 9
*N. oxiana*	− 13.33	− 15.26	2394	1.21	0.45	4.51	22	− 1	8
*N. philippinensis*	− 83.33	− 2.52	2072	0.13	0.00	0.03	139	♦	8
*N. samarensis*	− 83.72	− 2.59	2072	0.13	0.00	0.03	31	♦	28
*N. siamensis*	− 86.21	− 21.60	2077	1.10	0.02	0.15	− 11	− 2	− 7
*N. sputatrix*	− 89.29	− 3.60	2070	0.18	0.00	0.02	7	♦	− 7
*N. sumatrana*	− 28.37	− 8.64	2194	0.48	0.05	1.04	41	41	41

Gradual decrease in niche size and increase in human density are the threats on the Asian *Naja.*

^♦^As there was no suitable climatic niche, so the population could not be calculated.

**Figure 3:**
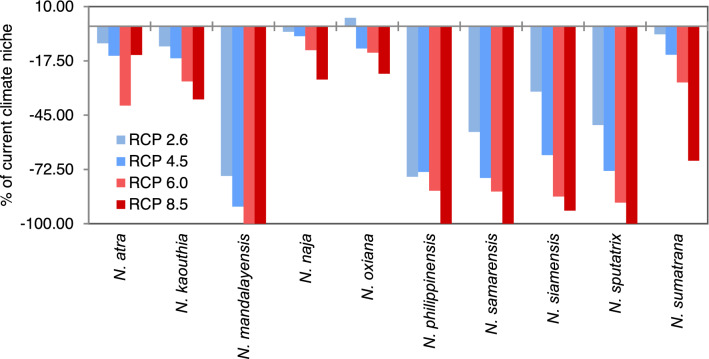
The reduction of the present climatic niche of 10 Asian cobras in four RCP trajectories in a timeframe of the next 50 years.

The analyses of future projections indicate that there will be a dramatic decrease in the potential climatic niche for every studied species within the next 50 years (Figs. 2 and 3). In the case of the Representative Concentration Pathway (RCP) 2.6 emission scenario, Asiatic *Naja* may lose averagely around 30% of their current climatic niche, which may be 66% in the worst scenario (RCP 8.5). In the same projection and timeframe, *N. naja* will remain at the top with the widest suitable niche, while *N. mandalayensis*, *N. philippinensis*, *N. samarensis,* and *N. sputatrix* may have no suitable climate space left. Depending on the different RCP trajectories, central Asia, northwest and central India, and Borneo Island might be climatically unsuitable for any cobras in the near future (Fig. 2 and Supplementary Figure).

A realistic prediction of climate change (RCP 6.0) indicates that an average of 56% (12–100%) of the area of suitable climatic niches of the studied species will be unsuitable within the next 50 years. In this scenario, the four species with the smallest niche size may also have no suitable climatic niche in their current range (Table 1). Other cobras distributed in the tropical climate of mainland and insular Southeast Asia (*N. kaouthia, N. siamensis*, *N. sumatrana, N. sputatrix*) will also lose half a portion (range 27–89%) of their current climatic niches. Considering the gradual process of climate change, the top three cobra species will be losing 1500 to 4000 square kilometres of suitable climatic niche annually. Through a similar process of niche loss, the remaining seven cobra species will be left with an average of < 1,000,000 square kilometres of potential niches in only a few decades (Table 1), which may eventually become climatically unsuitable within this century.

For each species, we combined the present and future climatic niches in RCP 6.0 and marked three zones to further understand niche dynamism (see “Methods”). For all cobra species, the amount of gaining climatically suitable niche space (Zone 2 in Table 1) is always smaller than the amount of losing a suitable niche, which is around one million square kilometres (Zone 1). On the other hand, only one-tenth of that amount of a new area (zone 2) will be climatically suitable for these cobras within the next five decades.

We generated the first climatic range maps for the ten species considered in our study. For each species, we analysed human populations in three different zone projections under an RCP 6.0 scenario. According to human demography projections, the habitat of *N. atra* will experience the greatest decline in human density by 2070. Also, *N. naja, N. kaouthia* and *N. siamensis,* will experience a decrease in human population by 2070, which may result in decreased human-induced pressure on the habitat and, consequently, on snake populations (Tables 1 and 2). On the other hand, *N. philippinensis* will face the greatest increase of human populations during the same time frame (Table 1). This species will have a combined impact of human population per square kilometre in zone 1 (> 395 in 2020 to > 534 in 2070) and zone 3 (~ 105 in 2020 and ~ 113 in 2070) along with a gradual decrease in niche size in zones 2 and 3. Other insular species may also encounter increased human densities in their current niches.

**Table 2:** Binary assessment denoted by + or − (present or absent) of some quantifiable selection pressures that currently affecting Asian cobras.

Species	Population decrease	Climate change			Anthropogenic activities			
		Decrease suitable niche	Influence of temperature	Influence of precipitation	Increase human density			Overexploitation and trade
					Zone 1	Zone 2	Zone 3	
*Naja atra*	+	+	+	+	−	−	−	+
*N. kaouthia*	+	+	+	+	+	−	−	+
*N. mandalayensis*	+	+	+	+	−	♦	♦	+
*N. naja*	+	+	+	+	+	−	−	+
*N. oxiana*	*	+	+	+	+	−	+	+
*N. philippinensis*	+	+	+	+	+	♦	+	+
*N. samarensis*	*	+	+	+	+	♦	+	+
*N. sagittifera*	*	*	*	*	*	*	*	+
*N. siamensis*	+	+	+	+	−	−	−	+
*N. sputatrix*	*	+	+	+	+	♦	−	+
*N. sumatrana*	−	+	+	+	+	+	+	+

^♦^As there was no suitable climatic niche, so the population could not be calculated.

*No published information.

**Figure 4:**
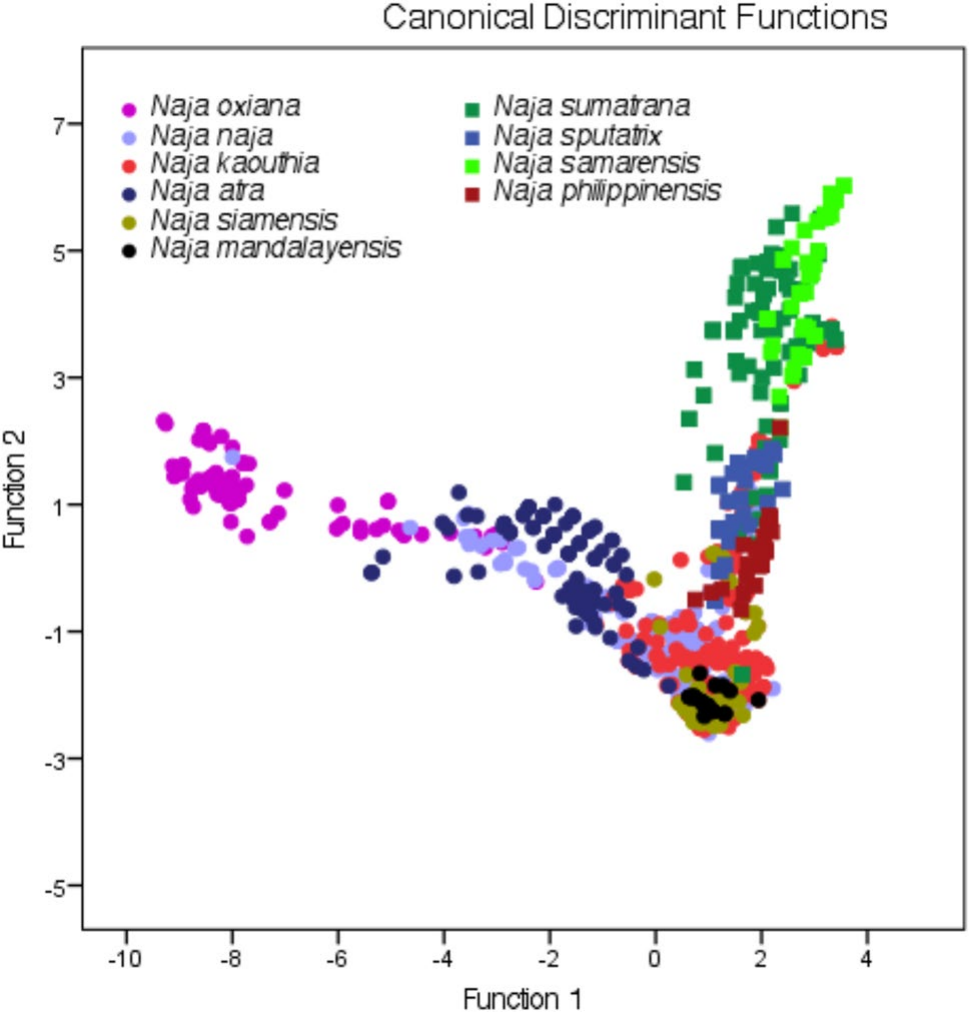
Clusters of 1162 occurrence points of ten *Naja* species. The solid circle is presenting species mostly distributed in Mainland and solid squares are presenting species distributed in Insular Southeast Asia.

**Figure 5:**
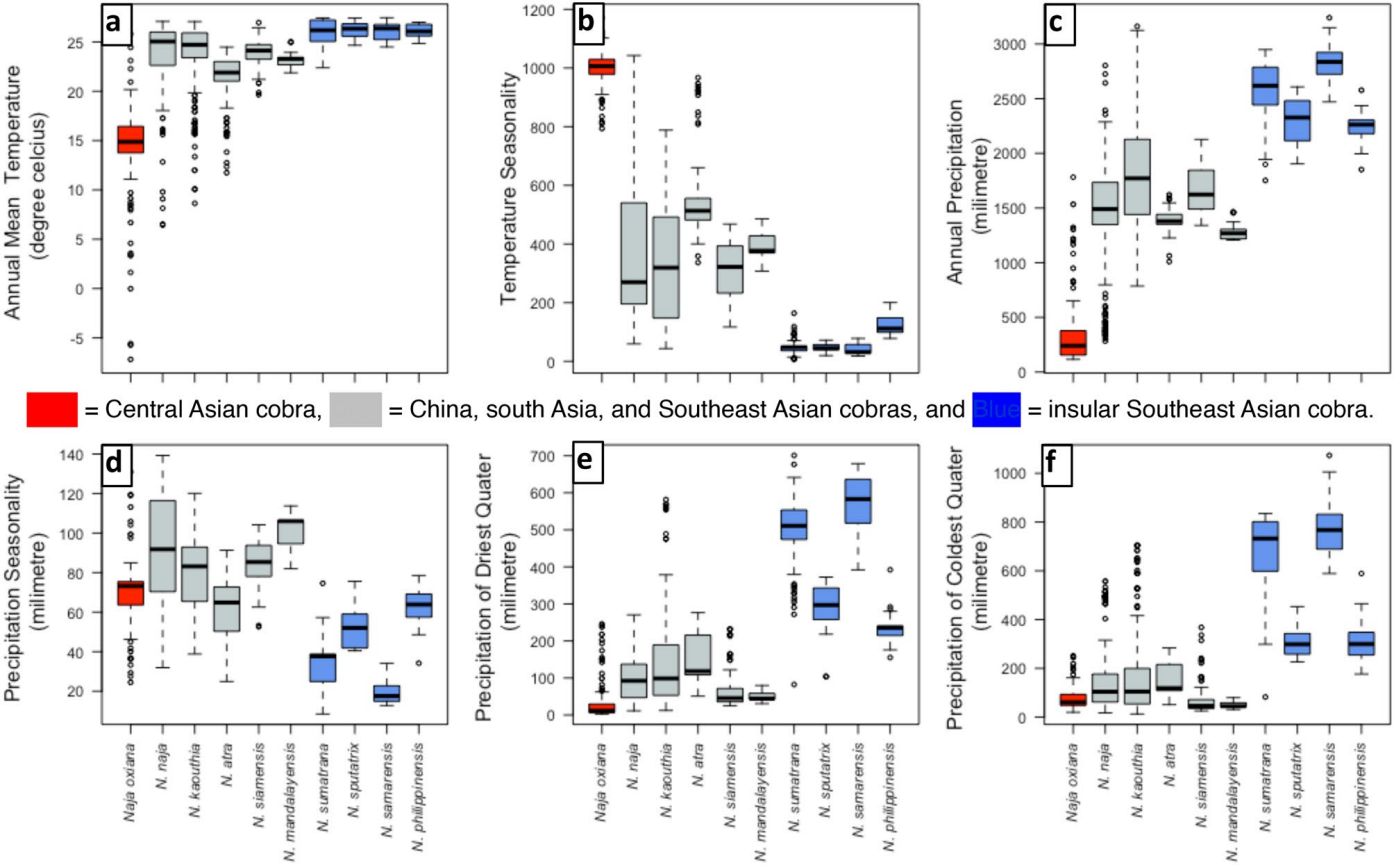
Six bioclim variables explain 74% of the cumulative variance to differentiate the three major climatic niche of Asiatic cobras. (**a**) Annual mean temperature, (**b**) temperature seasonality, (**c**) annual precipitation, (**d**) precipitation seasonality, (**e**) precipitation of driest quarter, (**f**) precipitation of coldest quarter.

Dimension reduction analysis reduced 13 bioclimatic variables of the occurrence points (n = 1162) into two principle functions that portray 74.3% of the cumulative variance (Function 1: 47.1%; Function 2: 27.2%) of the variables. The clustering of the observation indicates climatic niches for cobra species in the arid climate of central Asia, in tropical insular Southeast Asia, and in the humid tropical and subtropical climate of south Asia, south China and Southeast Asia (Fig. 4). In the latter region, the overlap of the occurrence points reflects the adaptation of cobras to similar climatic niches. Our prediction shows that the influence of the current climatic niche is mostly discriminated by two temperature and four precipitation variables (Fig. 5). Analysis on the variables of the occurrence location indicates that *N. oxiana* adapted to the highest fluctuations of diurnal temperature (13.98 ± 5.34 °C) in the dry climate region, while *N. philippinensis* adapted to the lowest fluctuation (26.12 ± 0.66 °C) of this variable in the humid climate region (Fig. 5a). Seasonality of temperature (bioclim 4) and annual precipitation (bioclim 12) subdivided the occurrences into three distinct clusters (Fig. 5b,c). Correspondingly, seasonality of temperature is high in central Asia and low on the islands. With a dry climate, central Asia is less isothermal than the tropical islands. Precipitation seasonality and precipitation in the winter season mainly define cobra niches on the tropical islands (Fig. 5d–f), as islands receive a greater amount of precipitation annually (> 2000 mm) and during the driest quarter (> 300 mm) of the year, which is usually winter, than in other parts of the study area (Fig. 5c,e). Mandalay is a dry environment in Myanmar and hosts an endemic cobra, *Naja mandalayensis*, which has an extremely restricted distribution^32^. The habitats of this species also receive more rain (> 1000 mm) than those of the other cobra species adapted to the dry environments of central Asia, *N. oxiana* (< 400 mm).

## Discussion

Our current climatic niche maps strongly resemble the known distributions of Asian cobras^13,14,16,32–34^. The climate of Southeast Asia is suitable for five Asian species of *Naja*: *Naja atra*, *N*. *kaouthia*, *N. mandalayensis*, *N. naja*, and *N. siamensis* (Fig. 1b–e,i). The widest favourable climate for *N. naja* is bordered by the Hindu Kush, Karakorum and Himalayas in the west and north, respectively. The climatic niches of this species and *N. oxiana* may overlap in the eastern part of the Hindu Kush Mountains^16^. Our results also suggest that the niche of *N. atra* is hindered to the Southwest by the Arakan—Naga mountain range on the China-Myanmar border, which is considered a distribution barrier also for other terrestrial elapids^35^ (Fig. 1b). Also our predictions for Southeast Asian insular cobras are consistent with their currently known distribution^16^ (Fig. 1g,h,j,k). Considering the similarities between the current occurrence range and climatic niche prediction, the future potential climatic niche may correspond to the future occurrence range of the species, if the species can disperse to the predicted ranges.

By mapping suitable climate, we found that the range of each cobra species will decrease in the future, while its fragmentation will increase and shift towards the east. Based on the occurrence records, the predicted future ranges seem to be accessible for all the mainland species^16^. However, cobras adapted to the islands of maritime Southeast Asia could survive both on neighbouring islands and on the mainland, but the ocean will act as a large barrier, potentially preventing successful niche tracking as temperatures increase in the near future.

We wish to add that some caution must be exerted when predicting future evolutionary scenarios with climate-envelope models, because by definition, this approach determines only the climatically suitable geographic space. The fundamental niche of a species, however, might be larger than the reconstructed climatic niche^36^, an aspect especially relevant for island species, which live in a very limited environment that most certainly does not reflect the true ecological capabilities of its inhabitants. In addition, genetic accommodation and assimilation of plastic phenotypes also need to be accounted for when predicting future effects of environmental change^37^.

A previous study on South American tropical vipers showed that habitat fragmentation and population isolation drastically changed predator pressure and food availability, resulting in major demographic changes^38^. Considering the current declining population trend of the studied species (Table 3), it may be assumed that population shrinkage and range fragmentation due to climate change may further accelerate extinctions across populations.

**Table 3:** Current conservation status and threats of 11 Asian cobras extracted from IUCN^17^ database.

English name	Chinese cobra	Monocled cobra	Burmese spitting cobra	Indian cobra	Central Asian cobra	Philippine cobra	Peters' cobra	Indo-Chinese Spitting ccobra	Indonesian spitting ccobra	Equatorial Spitting cobra	Andaman cobra
Scientific name	*Naja atra* Cantor, 1842	*Naja kaouthia* Lesson, 1831	*Naja mandalayensis* Slowinski and Wüster, 2000	*Naja naja* (Linnaeus, 1758)	*Naja oxiana* (Eichwald, 1831)	*Naja philippinensis* Taylor, 1922	*Naja samarensis* Boulenger 1896	*Naja siamensis* Laurenti, 1768	*Naja sputatrix* Boie, 1887	*Naja sumatrana* Müller, 1887	*Naja sagittifera* Wall, 1913
Last assessed in	2011	2011	2011	2011	1996	2007	2007	2011	2011	2011	*
Population trend					*		*		*		*
Current Red List status	VU	LC	VU	*	DD	NT	LC	VU	LC	LC	*
Previous status	LC	LC	*	*	*	*	*	LC	*	*	*
Altitude (m)	1630	1000	*	*	2100^♦^	800	800	*	600	1500	*
Main threats	Overexploitation	Overexploitation	Overexploitation	*	*	Trade	Trade	Trade	Trade	Trade	*

*No published information.

^♦39^.

*N. naja,* which occurs from the semi-arid environment of the Indus valley of Pakistan to tropical Southeast Asia, will lose the least amount (~ 12%) of its currently suitable climate. *N. oxiana* may disperse towards the east, which might be on the edge of its niche^39^. However, within this century, five of the Asian *Naja* species might meet the Red List assessment criteria B1(a)(b)(c) for Vulnerable status, which is less than 20,000 square kilometres of discontinuous extent of occurrence^40,41^. Analysing the rate of climatic niche shrinking, we found that the amount of habitat gain (zone 2, Table 1) is one-tenth of the amount of habitat loss (zone 1, Table 1). To mitigate this threat, high altitudes may provide a retreat with low temperatures more suitable for these cobras^42^. As most of the species have shown adaptations to high altitude (Table 3), the migration towards new suitable habitats may not be hampered by low elevation in South and Southeast Asia. The success of this expansion of populations to new geographic areas will be challenging due to the low dispersal abilities of snakes^43^, and in case of a rapid change, both the adaptability and survivability of the different species may be challenged^44^, as new climatically suitable niches might not provide suitable habitats with sufficient food and shelter. In addition to structural habitat features, tolerance and resilience of cobras might be further challenged by climate change^45^. Temperature and precipitation seasonality play a major role in discriminating distribution ranges, and these variables, along with precipitation^31^ during the driest and coldest quarters, largely differentiate the niches of Asian cobras. Except for *N. oxiana* and *N. atra*, the remaining mainland species share a common climate space, and our prediction suggests that Southeast Asia will become a shared habitat for these four species (Figs. 1 and Supplementary Figure). This may cause a strong competition for resources and result in competitive exclusions given these cobras display largely similar ecologies.

Along with small geographic range size, high levels of human exploitation were found to be important in determining the extinction probabilities of snake species^46^. Many different species of elapid snakes are being collected from the wild and traded across borders of various Asian countries^22,47^. Growing human populations may accelerate the exploitation of *N. oxiana*, *N. naja*, *N. samarensis*, and *N. philippinensis*, which are at the top of the trade list in the CITES database. In addition, the same database shows that a total of 24 different body parts extracted from *Naja* species were recorded in the import and export trade^47^. From the year 2000 to 2018, an average of 0.18 million of these items were traded on the international market, including 15,400 whole specimens of cobras, which were collected from various Asian countries, and most of them from the wild. China alone has a high demand for various snake species for traditional medicine, and a three-year study showed that about 13% of the 2.29–29.32 tons of wildlife are being traded every day on the Guangxi border between China and Vietnam, is snakes and snake stuff. The implementation of the CITES initiative and country-specific strategies against illegal trade has not been successful in stopping this overexploitation^47^.

The rapid economic development in our study area is accompanied by considerable exploitation of natural resources leading to environmental degradation^48^. In South Asia, 73% of the total land area is under agriculture, and almost half of the land area of Southeast Asia is in agricultural use^49^, and countries of this region will more than double their cultivated areas in the near future^48^. Furthermore, most of the agricultural lands currently in use are found in the areas of former primary forests, grasslands and wetlands; a conversion that resulted in the loss of biodiversity associated with those natural habitats^50,51^. Studies on growth patterns based on simulated future urban maps indicate that in Asia, fragmented or diffused patches of built-up lands will eventually connect and result in more aggregated urban landscapes^52^. Also, it is expected that the urbanization rate across the region will reach 50% by 2026^21^. Due to rapid urbanization, about half of the human population of Southeast Asia is predicted to inhabit urbanized regions by this time. Except for *N. atra*, and *N. siamensis*, Asiatic cobras may consequently lose their habitat in an even faster way via the combined impacts of decreasing suitable climate space and increased human population density (Table 2). Extension of protected areas over climatically suitable space may ensure uninterrupted habitats for cobras to withstand human pressure^10^.

It has already been suggested that public education and human welfare may be the best approach for successfully achieving conservation goals^53^. In 2020, however, the Sustainable Development Goals (SDG) meeting of the United Nations summarized the failure of all 20 SDGs^54^. Referring to this point, we consider RCP 6.0 to be a realistic scenario for 2070. Whereas our study is restricted to climate change and human populations, the future inclusion of other ecological, geographic, and environmental factors may produce more detailed predictions of the conservation status of Asian cobras.

Conflicts between cobras and humans may have significant impacts on public health. Some of the most densely populated tropical countries are host to the five deadliest cobra species along with several highly venomous species of krait (*Bungarus* spp.) and vipers in Southeast Asia, and thus a notable portion of global snakebite mortality occurs in this region every year, particularly affecting poor human populations^55^. Snakebite management should focus on this region to achieve the strategic goals to minimize this neglected tropical disease impact by 2030^56^. Our results show that the future geographic ranges of cobras might lead to an increase in snakebite incidents in new areas (zone 2, Table 1), e.g. higher elevations^55^. The potential migration of cobras to newly suitable climatic zones might require revisions to snakebite management plans and related investment in antivenom design, and our predicted future distributions of cobra species might act as a guideline to identify relevant “hot zones” for setting up surveillance programs. Respective countries may also upgrade their existing operations on antivenom production by considering the predicted future cobra distributions, thereby also accounting for inter-and intra-specific variation. Considering the influence of temperature and precipitation on the current occurrence of Asiatic cobras (Fig. 5) and on venom composition^57^, the efficacy of current antivenoms should be regularly tested under changing climate scenarios.

In conclusion, our study reveals that Asian cobras are threatened by climate change and human population pressure. Climate change will result in the reduction of both niche size and geographic distributions of the Asian mainland cobras, with predicted shortages in climatically and physically suitable habitats within a few decades. In addition, rapid transformation of land use will further impact accessible habitats. Southeast Asia is a suitable zone for five species of cobras but is predicted to undergo dramatic increases in natural habitat degradation due to rapid land transformation and overexploitation. We suggest that increasing monitoring and local law enforcement may restrict trade, improve public awareness and curb overexploitation. In addition to conservation activities, we emphasize the design of antivenom that considers intraspecific variation of venom from different geographic regions to assist snakebite management, which would be a step forward in achieving the anticipated goals of the World Health Organization by the year 2030^25,56^.

## Materials and methods

### Data on species distribution

Ten Asian cobra species were considered in this study. Of the ten studied species, six are mostly distributed on the mainland and the remaining four are restricted to maritime Southeast Asia^14,58^. The preliminary database on the geographical distribution of ten *Naja* species was developed from the scientific literature^32–34,39,59–63^ and the collection records of *Naja naja* and *N. kaouthia* at the Venom Research Centre, Bangladesh. Additionally, literature and preserved specimen based occurrence records were collected from the GBIF database (www.gbif.org, accessed on 17.11.2020), and research-grade and verifiable data were collected from iNaturalist (www.inaturalist.org, accessed on 17.11.2020) and from IUCN Red List range map (https://www.iucnredlist.org, accessed on 17.11.2020). Finally, duplicate and confusing occurrence points were filtered out based on the scientific publications^13–16,32,33^ and the Reptile Database^11^. In case of multiple points from a small area, the closely positioned points were filtered out to minimize the bias of the model. After scrutiny, 1162 points of ten Asian cobra species were selected to determine the climatic niche (Supplementary dataset). We used 111 occurrence records of the Central Asian cobra (*Naja oxiana*), 354 of the Indian cobra (*N. naja*), 218 of the Monocled cobra (*N. kaouthia*), 128 of the Chinese cobra (*N. atra*), 76 of the Indo-Chinese Spitting cobra (*N. siamensis*), 29 of the Burmese spitting cobra (*N. mandalayensis*), 123 of the Equatorial Spitting cobra (*N. sumatrana*), 54 of the Indonesian cobra (*N. sputatrix*), 41 of the Peters' cobra (*N. samarensis*), and 28 of the Philippines cobra (*N. philippinensis*) to represent current distributions in Asia (Fig. 1a).

### Study area

A cropped rectangle of Asia (Longitude 40 to 150, and Latitude -10 to 50) covering the current distribution of Asian *Naja* species was used as the focus area in this study. The study area extends from Southern Kazakhstan and Mongolia in the north, excluding Australia in the South, from Japan and Papua New Guinea in the east, to Yemen in the west. The wrld_simpl () spatial polygon data frame from maptools^64^ package is used to generate maps of this study.

### Climate variables

Bioclim variables for current and future (year 2070 ) climate were obtained from a set of 19 bioclim raster files (0.5° × 0.5° resolution) downloaded from ecoClimate (www.ecoclimate.org, accessed on 17.11.2020)^65^. We have used four Atmosphere–Ocean General Circulation Models (AOGCMs): Community Climate System Model (CCSM), Centre National de Recherches Météorologiques (CNRM), Model for Interdisciplinary Research on Climate (MIROC) and Meteorological Research Institute (MRI). Bioclim variables were extracted from 0.5° terrestrial quadrate (= 60 × 60 square kilometre) of the raster of each model within the study area (n = 24,000 quadrates) and all occurrence points (n = 1162) for current and future climate scenarios.

### Correlation

As the variables have different measuring units, observations were standardised (z scored). To avoid multiple collinearities and bias, the Pearson correlation coefficient > 0.9 was used to omit five highly correlated bioclimatic variables (bioclim 5, 6, 7, 11, 14, and 16). Minimum temperature of the coldest month (bioclim 6) and the mean temperature of the coldest quarter (bioclim 11) were removed to reduce possible bias on annual mean temperature (bioclim 1) in Asia. Temperature annual range (bioclim 7) is derived from the maximum temperature of the warmest month (bioclim 5) and minimum temperature of the coldest month (bioclim 6) and is highly correlated with temperature seasonality (bioclim 4); this was also removed. Mean temperature of the warmest quarter (bioclim 10) is more general than the max temperature of the warmest month (bioclim 5) and was therefore selected. Precipitation of the wettest quarter (bioclim 16) is mostly dominated by the Monsoon, which is a key contributor to annual precipitation (bioclim 12) and precipitation of the wettest month (bioclim 13). Bioclim16 was removed in favour of bioclim 12. Precipitation of the driest quarter (bioclim 17) is more general than precipitation of the driest month (bioclim 14) and hence bioclim 17 was selected over bioclim 14. After filtration, a total of 13 dimensions (bioclim variables 1, 2, 3, 4, 8, 9, 10, 12, 13, 15, 17, 18, 19) of two vital abiotic factors (temperature and precipitation) were selected for the study.

### Ecological niche models

Ecological niche models (ENMs) have been widely applied to predict the effect of climatic change on the distribution of a species with contextual information and interpretation^66^. Algorithms, such as Bioclim, applied on the climatic variables of the presence-only data of a species generate a projection of the climatically suitable areas for that species^67^. Bioclim maps the multivariate climatic suitable niche of a species (percentile distribution of the data in the n-dimensional climatic space), and thus the areas inside the climatic tolerance sampled for the species.

### Raster analysis

Raster analysis was performed in R using the libraries raster^68^, maptools^64^, rgdal^69^, and sp^70^. With these 13 selected variables, the *bioclim()* function of the Dismo^71^ package was trained. The *predict()* function was then used to predict the suitable climatic niche of each species in the study area. A consensus map was generated by averaging predicted values of the identically positioned grid from four Atmosphere–Ocean General Circulation Models using *mosaic()* function. Assuming that the consensus model will enhance uncertainty and error associated with the different simulations. All four greenhouse gas concentration trajectories corresponding to the Representative Concentration Pathway (RCPs) have been used to estimate the future climate. For estimates of future climate size and human population, we used a moderated emission scenario RCP 6.0 that represents an optimistic context^10^. All R-packages used in this study are licensed for open access (https://www.gnu.org/licenses/gpl-3.0.html).

### Geographical shift of potential niche

The prediction values were set to 0 (− inf to 0) and 1 (0 to + inf) in the case of present climate, and 0 (− inf to 0) and 2(0 to + inf) in the case of 2070 climate. Overlapping these two rasters of each species creates three zones of potential niche: zone 1 (value 1, orange in Supplementary Figure) represents areas that are currently suitable for the respective species and will be unsuitable by the year 2070; zone 2 (value 2, light green) represents the area that is not currently suitable but will be suitable by the year 2070; and zone 3 (value 3, dark green) is the area that is currently suitable and will remain so for the next 50 years. Most of the Asian cobra records come from several hundred metres of elevation^15,72,73^ and hence their potential climatic niches in small hill terrain were considered in our study. Suitable but inaccessible (due to water barrier, distant location from the current range, mountain range etc.) climatic spaces were masked off by using the *mask()* function^68^. Niche size change was calculated from the suitable and accessible climate spaces. We calculated the time for each *Naja* species to reach a vulnerable state in the category of geographically accessible niche, which was projected based on the average amount of area that will be unsuitable per year. The size of the climate niche was then assessed by following the Red List criteria.

### Human population

Global total population projection rasters based on Shared Socioeconomic Pathways (SSPs) at a resolution of 30-s were downloaded from Socioeconomic Data and Applications Center (SEDAC, https://sedac.ciesin.columbia.edu on 19.10.2019). The rasters were downgraded to 0.5° × 0.5° resolution using *aggregate()*^68^ function by summing the grid with the factor of 60 × 60 and then cropped to the study area^74^. SSPs describe alternative future trends of societal factors (such as demographics, economics, technological development, governance, etc.) that can be combined with climate projections to carry out integrated analyses^75^. Only the accessible and suitable areas of the climatic extent of occurrence, of the present and 2070, for each species, were used as a reference map to obtain the human population. We used human population density (number per square kilometre) for our analysis.

### Risk assessment

Three zones of the climatic niche, described in the Raster analysis subsection, were considered to assess the risk of threat on the climatic niche size of the studied cobra species. Niche size and human population in every niche zone were obtained for the present and the future (the year 2070). We assumed that the more the human populations are in a zone, the more unsuitable the zone is for snakes. All available information on threats, such as population trends of each species, trade, and exploitation was also added to the assessment of future risk.

## Supplementary Information

Supplementary Information 1.

Supplementary Information 2.
