# Supplementary material for: Climate change and the increase of human population will threaten conservation of Asian cobras
Source: Sci Rep. 2021 Sep 13;11:18113. doi: 10.1038/s41598-021-97553-4 (PMC8438023; doi:10.1038/s41598-021-97553-4)
Supplement: Supplementary file 2 — Supplementary Information 2. [file 41598_2021_97553_MOESM2_ESM.pdf]

Climate change and the increase of human population will threaten conservation of Asian cobras

Mohammad Abdul Wahed Chowdhury[1,2,3,4*], Johannes Müller[1,2], Sara Varela[1,5]

[1] Museum für Naturkunde, Leibniz-Institut für Evolutions- und Biodiversitätsforschung, Berlin 10115, Germany

[2] Institut für Biologie, Humboldt-Universität zu Berlin, Berlin 10115, Germany

[3] Department of Zoology, University of Chittagong, Chattogram 4331, Bangladesh

[4] Venom Research Centre, Department of Medicine, Chittagong Medical College, Chattogram 4203, Bangladesh

[5] Centro de Investigación Mariña, Universidade de Vigo, Grupo GEA, Departamento de Ecoloxía e Bioloxía Animal, Vigo 36310, Spain.

* piloctg@yahoo.com

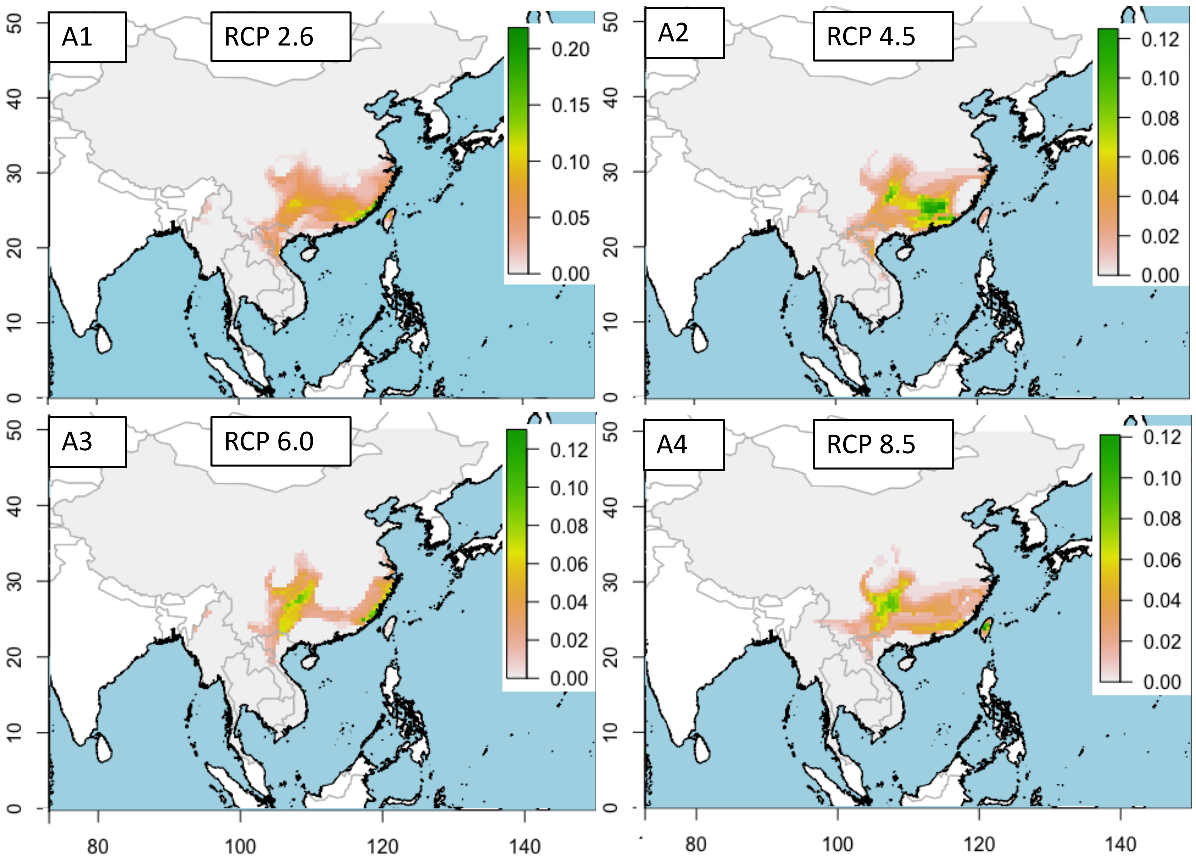

A1
RCP 2.6
A2
RCP 4.5
A3
RCP 6.0
A4
RCP 8.5
0.20
0.15
0.10
0.05
0.00
0.12
0.10
0.08
0.06
0.04
0.02
0.00
50
40
30
20
10
0
80
100
120
140

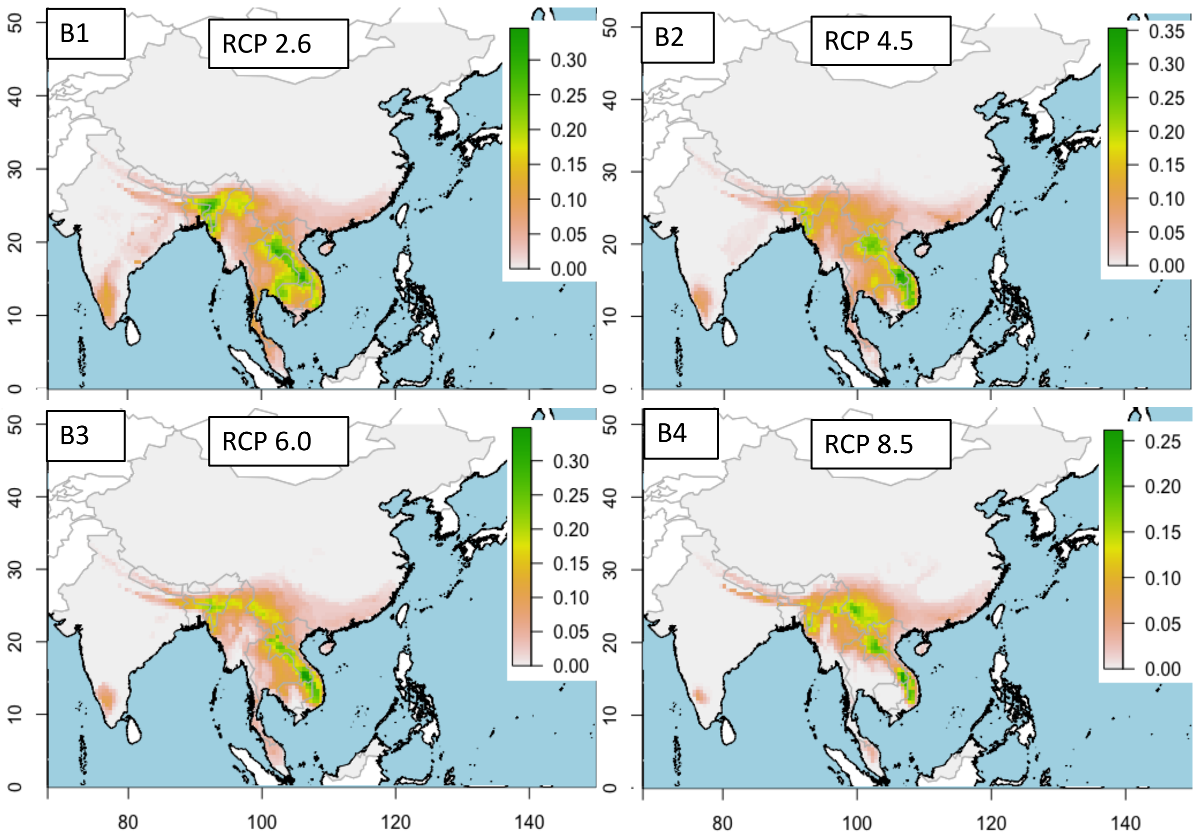

B1
RCP 2.6
B2
RCP 4.5
B3
RCP 6.0
B4
RCP 8.5

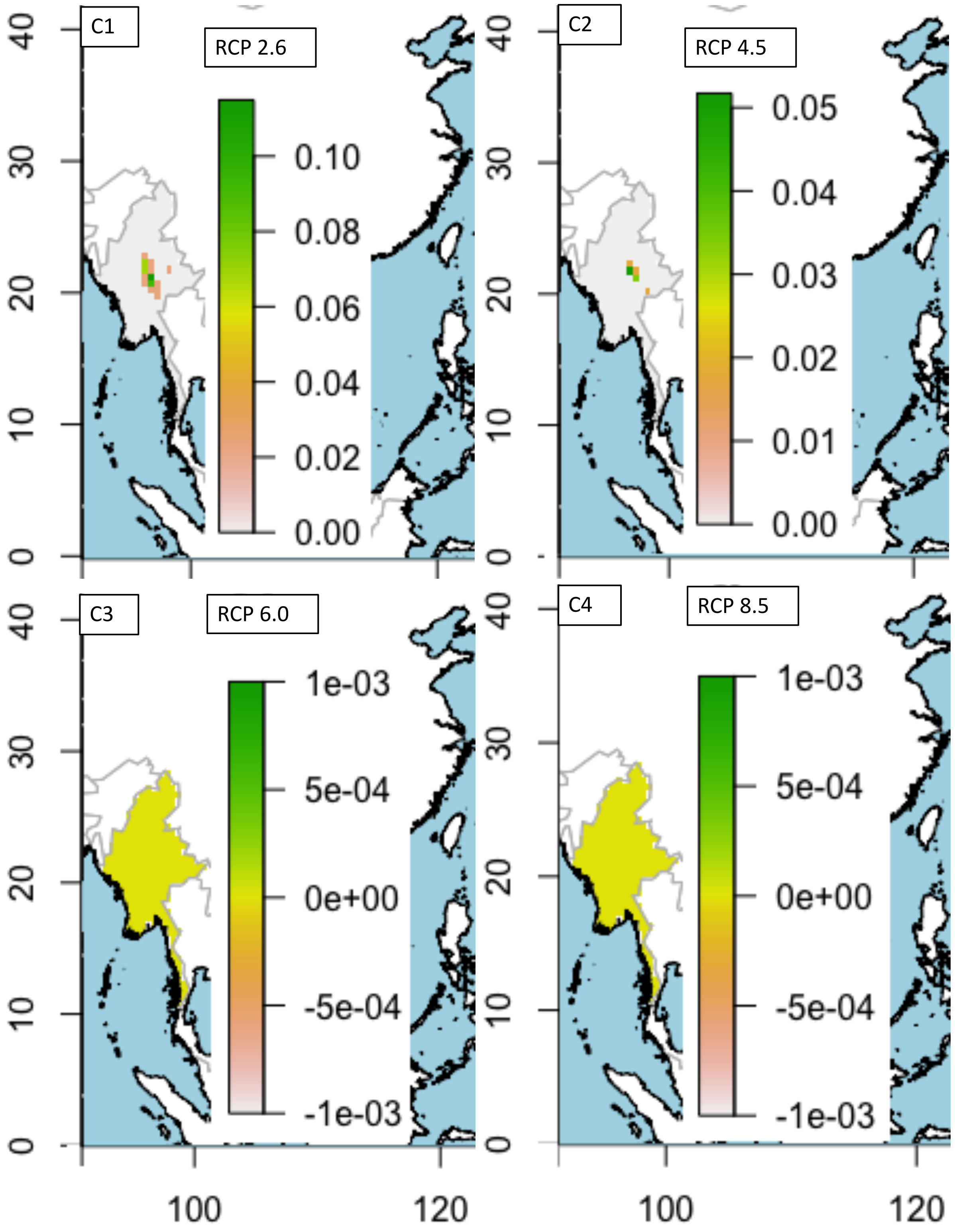

C1
RCP 2.6
0.10
0.08
0.06
0.04
0.02
0.00
C2
RCP 4.5
0.05
0.04
0.03
0.02
0.01
0.00
C3
RCP 6.0
1e-03
5e-04
0e+00
-5e-04
-1e-03
C4
RCP 8.5
1e-03
5e-04
0e+00
-5e-04
-1e-03
0
10
20
30
40
100
120

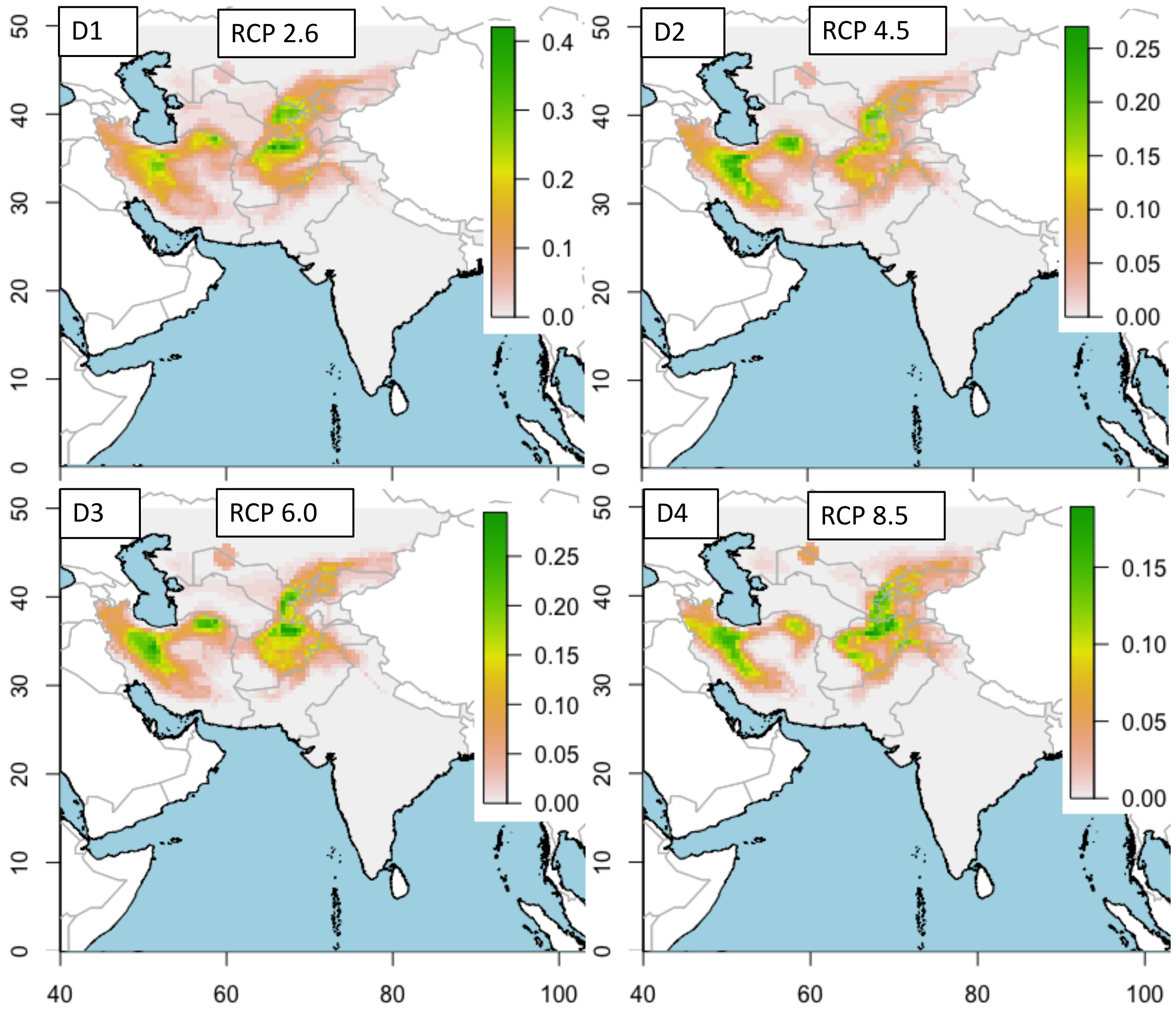

D1
RCP 2.6
0.4
0.3
0.2
0.1
0.0
D2
RCP 4.5
0.25
0.20
0.15
0.10
0.05
0.00
D3
RCP 6.0
0.25
0.20
0.15
0.10
0.05
0.00
D4
RCP 8.5
0.15
0.10
0.05
0.00
0
10
20
30
40
50
40
60
80
100

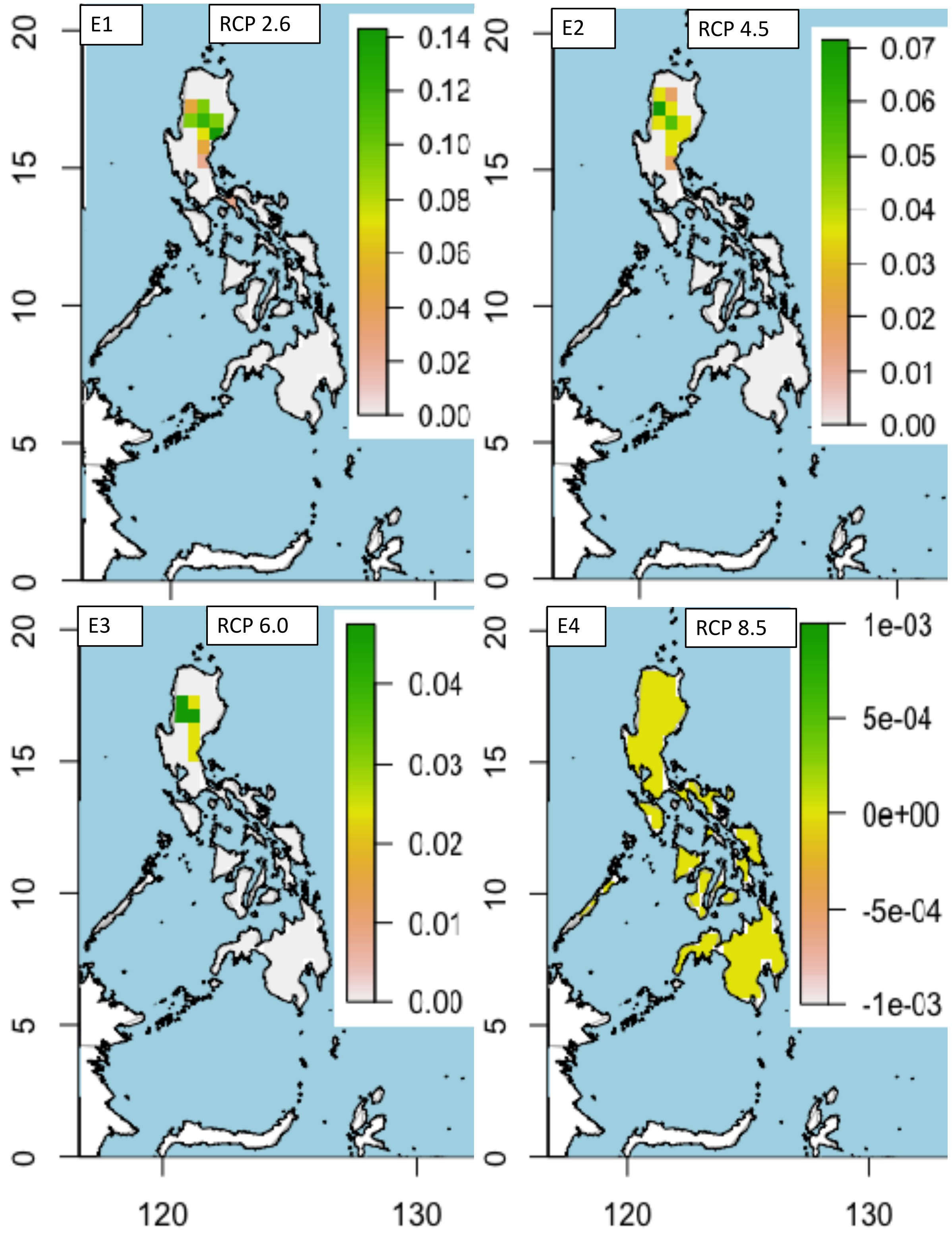

E1
RCP 2.6
0.14
0.12
0.10
0.08
0.06
0.04
0.02
0.00
E2
RCP 4.5
0.07
0.06
0.05
0.04
0.03
0.02
0.01
0.00
E3
RCP 6.0
0.04
0.03
0.02
0.01
0.00
E4
RCP 8.5
1e-03
5e-04
0e+00
-5e-04
-1e-03
0
5
10
15
20
120
130

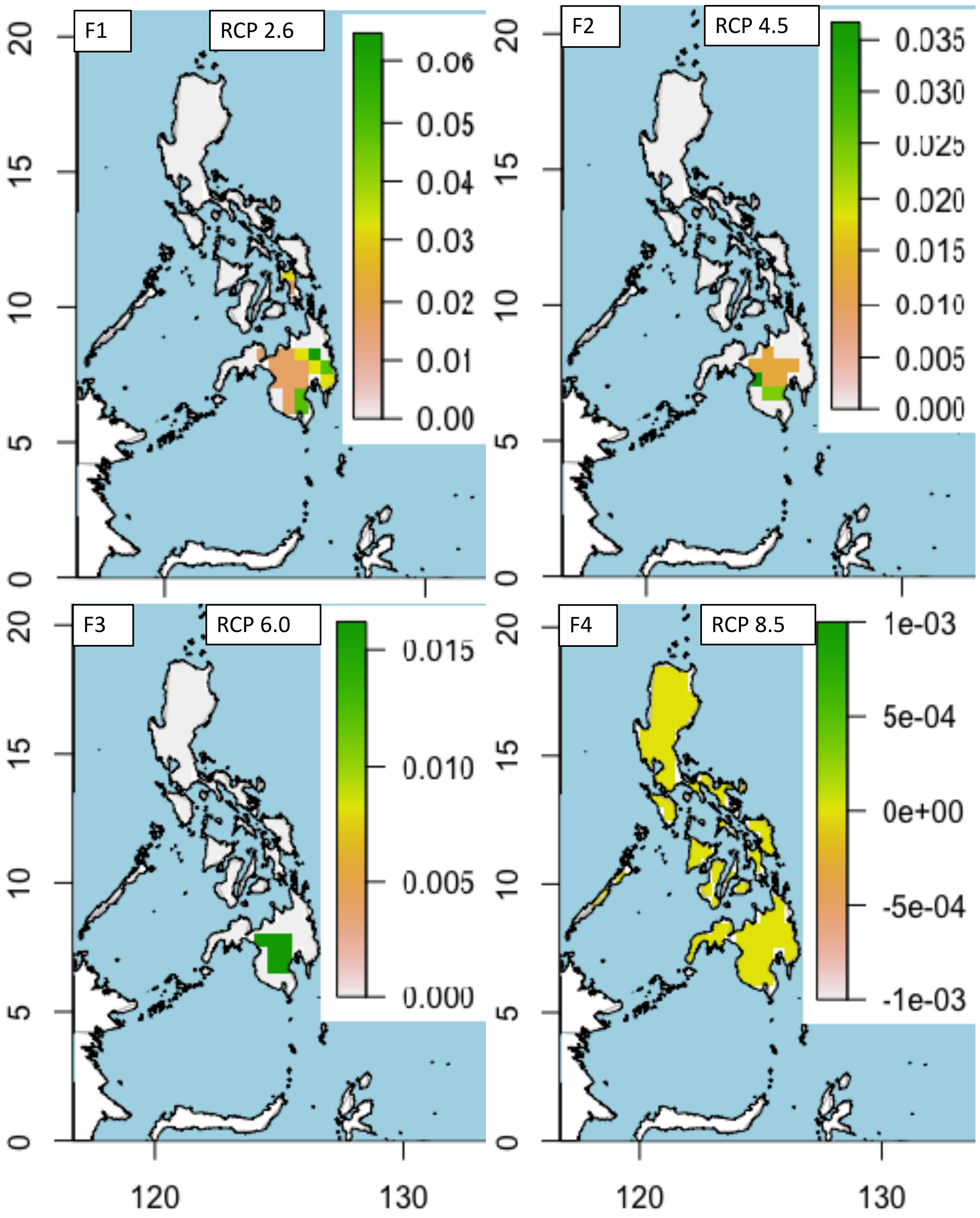

F1
RCP 2.6
0.06
0.05
0.04
0.03
0.02
0.01
0.00
F2
RCP 4.5
0.035
0.030
0.025
0.020
0.015
0.010
0.005
0.000
F3
RCP 6.0
0.015
0.010
0.005
0.000
F4
RCP 8.5
1e-03
5e-04
0e+00
-5e-04
-1e-03
20
15
10
5
0
120
130

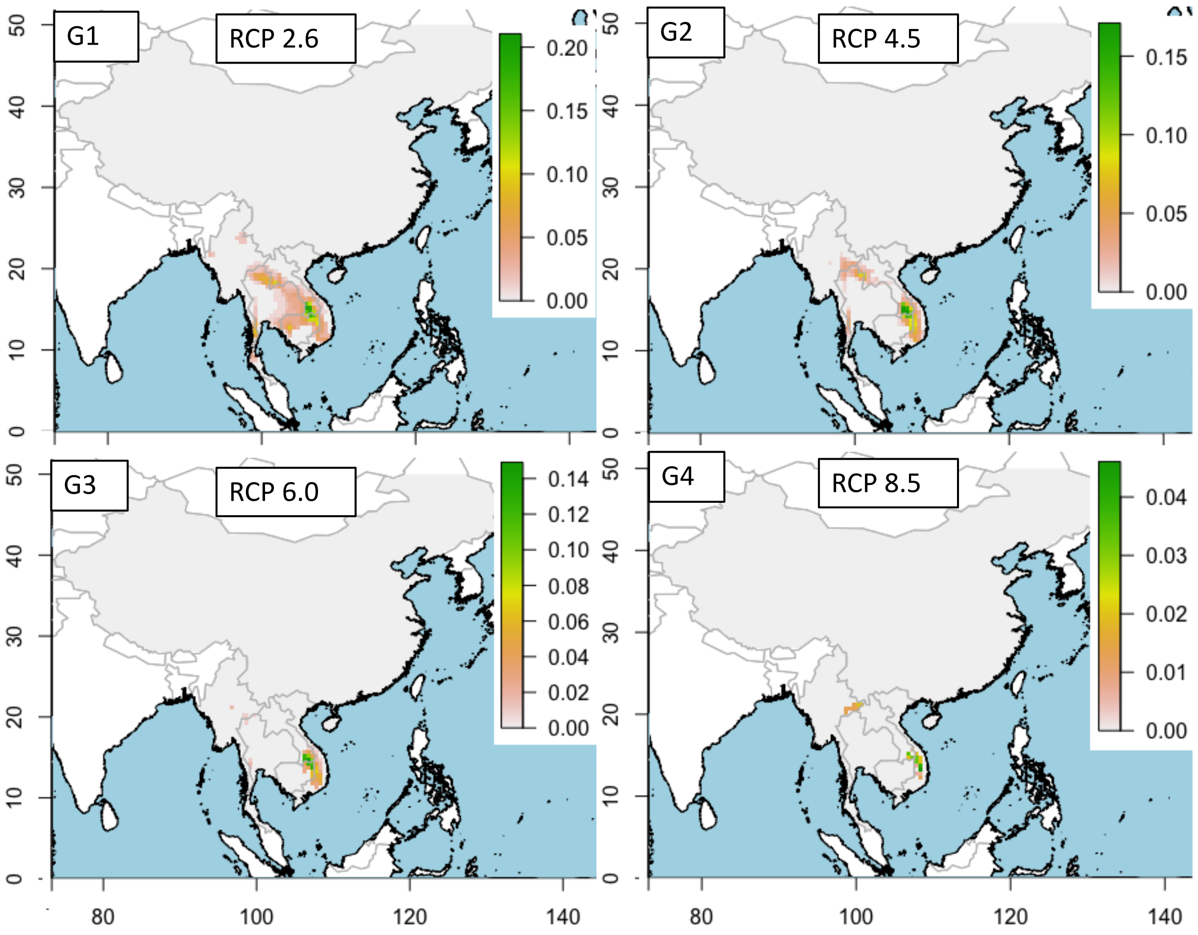

G1
RCP 2.6
G2
RCP 4.5
G3
RCP 6.0
G4
RCP 8.5
0.20
0.15
0.10
0.05
0.00
0.14
0.12
0.08
0.06
0.04
0.02
0.03
0.01
50
40
30
20
10
0
80
100
120
140

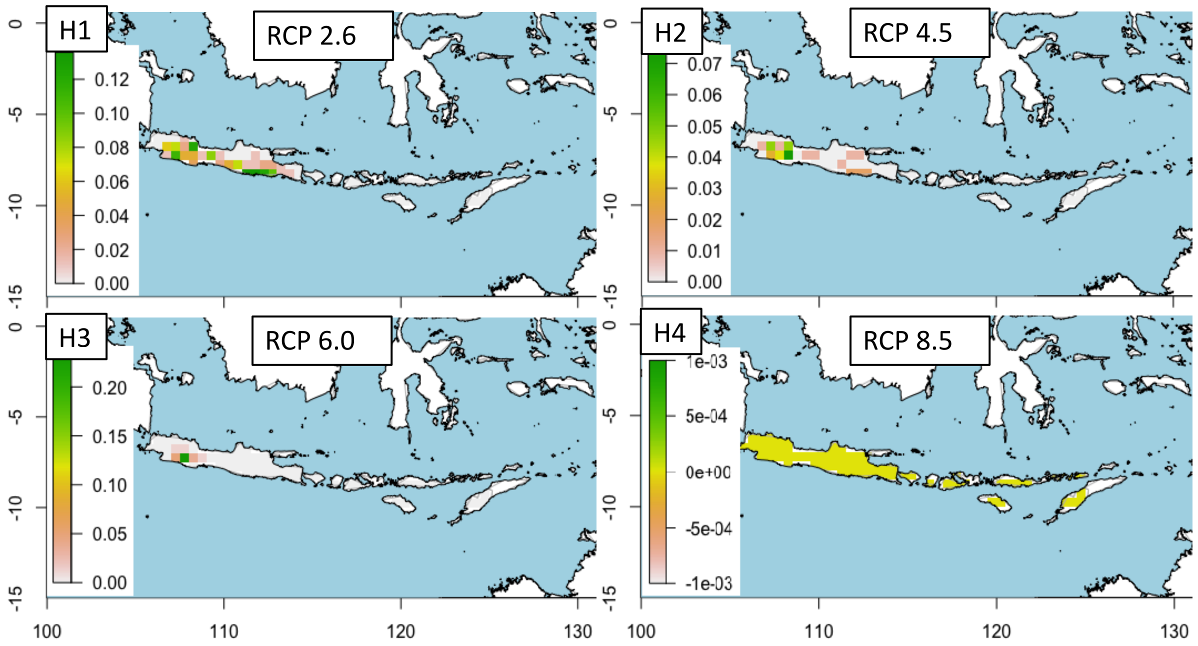
H1
RCP 2.6
0.12
0.10
0.08
0.06
0.04
0.02
0.00
H2
RCP 4.5
0.07
0.06
0.05
0.04
0.03
0.02
0.01
0.00
H3
RCP 6.0
0.20
0.15
0.10
0.05
0.00
H4
RCP 8.5
1e-03
5e-04
0e+00
-5e-04
-1e-03
0
-5
-10
-15
100
110
120
130

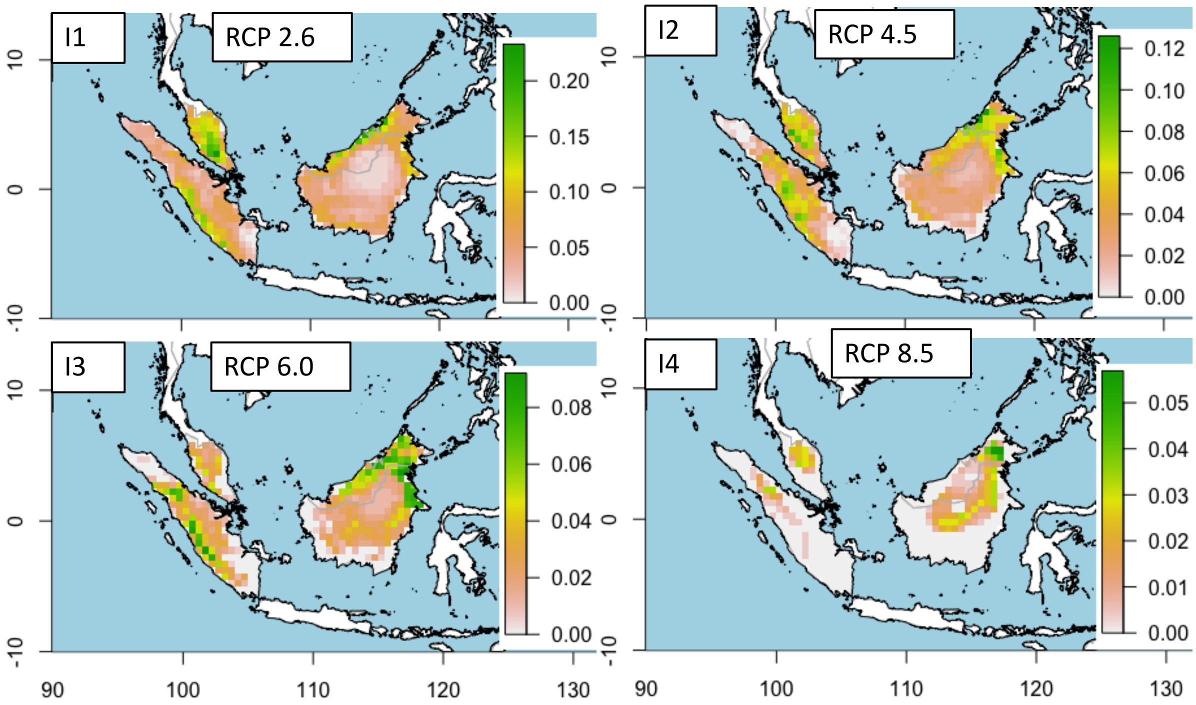

Supplementary figure: Consensus potential climatic niches for future (in the year 2070) climate scenario in four RCPs (2.6, 4.5, 6.0, and 8.5) of nine Asiatic *Naja* species (maps of *Naja naja* niche are given in the main text), (A) *Naja atra*, (B) *N. kaouthia*, (C) *N. mandalayensis*, (D) *N. oxiana* (E) *N. philippinensis*, (F) *N. samarensis*, (G) *N.* siamensis, (H) *N. sputatrix*,and (I) *N. sumatrana*. The maps were generated from spatial polygon data frame of wrld_simpl function of maptools[62] r-package.
